# Public perception and changing attitudes toward antidepressants over a decade in social media: Lessons learned from online discussion using artificial intelligence

**DOI:** 10.1371/journal.pone.0318464

**Published:** 2025-09-04

**Authors:** Min Ho An, Min-Gyu Kim, Jueon Kim, Seheon Chang, Dong Yun Lee, Rae Woong Park

**Affiliations:** 1 Department of Biomedical Informatics, Ajou University School of Medicine, Suwon, Republic of Korea; 2 Department of Medical Sciences, Graduate School of Ajou University, Suwon, Republic of Korea; 3 Department of Dentistry, Kyung Hee University School of Dentistry, Seoul, Republic of Korea; 4 CNS Department of Computer Science, University of Texas at Austin, Austin, Texas, United States of America; 5 Bongdam Forest Mental Health Clinic, Hwaseong, Republic of Korea,; 6 Center for Biomedical Informatics Research, Ajou University Medical Center, Suwon, Republic of Korea; Northwestern University Feinberg School of Medicine, UNITED STATES OF AMERICA

## Abstract

**Background:**

Antidepressants play a crucial role in treating mental health disorders such as depression and anxiety. Understanding of patients’ perspective on antidepressants is essential for improving treatment outcomes; however, year-to-year change in the public’s perception of antidepressants remains unclear. We aimed to analyze changes in public sentiments and predominant perceptions regarding antidepressants using artificial intelligence pipeline.

**Methods:**

This study analyzed online discussions related to antidepressants on Reddit from January 1, 2009, to December 31, 2022. Antidepressant-associated communities were explored to collect a list of discussions relevant to antidepressant therapy. Discussion topics on antidepressants were identified using BERTopic, and the sentiments were analyzed using a RoBERTa model. Trends were assessed using the Mann–Kendall test to evaluate shifts in sentiments over time.

**Results:**

We analyzed 429,510 antidepressant-related discourse over 14 years and found a predominance in negative sentiments. Key discussion topics include the benefits and side effects of antidepressants, experiences with drug switching, and specific concerns regarding bupropion therapy. In trend analyses, negative sentiments decreased, while neutral sentiments increased over time. This aligns with a decline in the annual proportion of topics associated with side effects within each cluster.

**Conclusions:**

Negative perceptions toward antidepressants are prevalent on social media, mainly focusing on efficacy and side effects. However, a decade-long analysis shows a decline in negative sentiments, with an increase in neutral sentiments with a downturn in yearly proportion of side-effected related topics within each cluster. These trends and information may help improve strategies to address barriers to antidepressant use and adherence.

## Introduction

Antidepressants are essential for managing and treating various mental health conditions, such as depression and anxiety, which are prevalent globally [1–3,4]. They are among the most frequently prescribed medications in the United States (US) from the age of 20 to 59 years [5,6]. Luo et al [7] have also documented a rise in the prevalence of major depressive disorder (MDD) among patients in the US, accompanied with an increase in the prescription of antidepressant medications [8].

Owing to their proven efficacy and large availability, antidepressants are crucial in the clinical management of MDD [9,1]. Despite the potential therapeutic benefits, many patients often hesitate in initiating or adhering to antidepressant therapy [10]. Adherence to long-term antidepressant therapy is crucial for symptom remission and relapse prevention in MDD [11]. For this reason, the importance of physicians’ education in preventing the early discontinuation of antidepressants have been emphasized [3] and integrated into clinical practice. In this context, a comprehensive understanding of patient-level perspectives on antidepressants is essential for improving treatment outcomes and developing public health strategies involving antidepressant use. While there have been studies on anti-stigma research related to antidepressants, much of the existing research is based on traditional survey methods and does not capture the evolving public sentiment over time, particularly in the context of modern digital platforms like social media.

Social media platforms have become valuable in acquiring public health perceptions beyond the clinic, [12,13] often used for sharing experiences, seeking advice, or sparking debates on health topics. For instance, prior research has harnessed social media as a data source to explore the public’s perspective on statins [14,15]. In recent years, Reddit, a popular social media platform, has become a significant hub for health-related discussions. Its structure, organized around user-created communities called “subreddits,” encourages open and often anonymous discussions on diverse topics, including personal experiences with medications. At the time of data collection for this study, Reddit offered relatively accessible data through its API, a key advantage over other platforms with more restricted access. The volume of user-generated content is also substantial; in 2022, the platform recorded an upload of over 490 million posts and 2 billion comments [16]. This provides an extensive dataset of public opinions, experiences, and concerns about various health topics, including antidepressants [17,18]. When analyzing such large volumes of social media data, leveraging artificial intelligence techniques such as natural language processing (NLP) can efficiently process, interpret, and extract valuable insights [19,15,20].

Therefore, we hypothesized that social media data drawn from Reddit, when analyzed using artificial intelligence, could reveal trends in public sentiment, common perceptions, and potential misconceptions about antidepressant therapy. This study aimed to analyze trends in public perceptions and concerns regarding antidepressants by examining the complete corpus of discussions in Reddit from 2009 to 2022, totaling up to 14 years of user discussions.

## Materials and methods

### Ethics approval and statement

Analyses were approved by local scientific and ethics committees (Ajou University Medical Center Institutional Review Board: AJOUIRB-EX-2024–258). Also, this study conformed to the guidelines of the Standards for Reporting Qualitative Research [21]. The data were analyzed anonymously.

### Data sources

This study used Reddit[21] as the data source. This platform consists of various communities, each designated by an “r/” prefix. Users can engage with Reddit by posting to start a new discussion thread or by adding comments on others’ posts within these discussions. Most of these communities, including all the posts and comments within them, are open to public viewing and access.

To curate a dataset of antidepressant-related discussions on Reddit, we employed the methodology delineated by Somani et al [15]. Initially, we identified appropriate communities by querying the terms “depression” and “antidepressant” in the platform’s search engine, subsequently selecting those communities recommended by the engine for both queries. All posts and comments including author information, name of subreddit, title, body of text, created time within these identified communities were systematically collected using the Pushshift Application Programming Interface (API). We collected data from 2009 to 2022: four years were filtered after the platform’s inception in 2005 for data quality management and all data available until the time of data collection was used. This search was conducted for case-insensitive occurrences of the term ”antidepressant” and for both the generic and brand names of specific antidepressants (S1 Table) [22].

### Data Preprocessing and Cleansing for the Text Analysis of Social Media Posts

**Fig 1:**
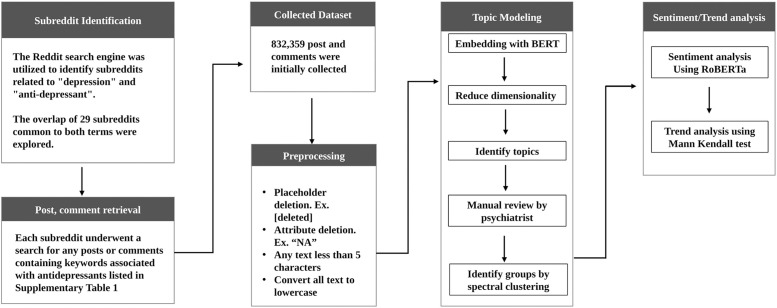
AI driven topic clustering and trend analysis workflow. The workflow for analyzing public perceptions of antidepressants on Reddit is illustrated. First, candidate subreddits discussing depression and antidepressants were identified using the Reddit search engine, revealing an overlap of 29 relevant subreddits. Each subreddit was then searched for posts or comments containing antidepressant-related keywords listed in Supplementary Table 1, resulting in an initial collection of 832,359 posts and comments. The text data underwent preprocessing, including placeholder deletion (e.g., [deleted]), attribute deletion (e.g., “NA”), removal of any text less than 5 characters, and conversion of all text to lowercase. Topic modeling was performed by embedding the text data with BERT, reducing dimensionality, and identifying topics, which were then manually reviewed by a psychiatrist and grouped using spectral clustering. Sentiment analysis was conducted using the RoBERTa model, followed by trend analysis using the Mann-Kendall test to evaluate shifts in public sentiment over time.

In the preprocessing of 832,359 collected raw text data, several steps were undertaken to ensure data quality and relevance (Fig 1). Initially, we identified and removed duplicate entries, resulting in the exclusion of 12,587 rows. Next, we eliminated rows containing null values (n = 15). Subsequently, instances of text indicating post or comment deletion, characterized by placeholders such as “[deleted]” or attributes such as “NA,” were replaced with empty spaces. Furthermore, any text comprising fewer than five characters was removed from the dataset (n = 18). The final stage of preprocessing involved converting all texts into lowercase for consistency. Ultimately, the dataset comprised 819,739 posts and comments.

### Topic modeling

For analyzing trends that reflect public perception, we first clustered users’ topics. Specifically, we employed BERTopic, [23] an advanced NLP method that transforms natural language into high dimensional vectors and clusters them. Various discussion topics surrounding antidepressants were identified using BERTopic. We first used the all-MiniLM-L6-v2 pretrained model [24] to embed the posts and comments into vectors in high dimensional space. These embeddings were first visualized using the uniform manifold approximation and projection (UMAP) technique, a dimensionality reduction technique. Next, we used HDBScan to cluster the vector embeddings of documents, thereby grouping the documents, in this case the posts and comments collected, into specific topics. Finally, we used class-based term frequency inverse document frequency (c-TF-IDF) techniques to identify keyword expressions for each topic based on the documents presented. Based on these representative keywords for each cluster, the topics that each cluster holds were identified.

Given that the topics generated by BERTopic are highly granular, we conducted another clustering to select large topics, as applied in a previous study [15]. Before additional clustering, each generated topic was manually reviewed by a psychiatrist to ensure accurate and appropriate large topic clustering. This process involved the examination of at least five documents, which were randomly selected from within each topic to warrant the relevance and consistency of the topic categorization. Subsequently, UMAP and spectral clustering were conducted on the c-TF-IDF representation of each topic to group these topics. To measure clustering performance and determine the final number of clusters, we used silhouette coefficient measures [25] and the Davies–Bouldin index [2].

### Sentiment and trend analyses

After topic clustering, we analyzed public’s sentiments from all documents and topics and their trends. Sentiment analysis techniques are designed to identify and assess the underlying tone or attitude present in relation to a specific topic within a document’s context [26]. The sentiment of each post was evaluated using a pretrained model, specifically a version of RoBERTa fine-tuned on a dataset for sentiment analysis. RoBERTa is a model trained with a wide range of publicly available dataset such as the English wikipedia, BookCorpus, CC-news. Its original publication does not list Reddit as a source of training data. [27] RoBERTa can assign multiclass labels to text, categorizing it as positive, neutral, or negative. This model has gained recognition in recent research focused on healthcare issues, particularly in studies that analyze data derived from social media platforms [28,29,15]. For each discussion, RoBERTa calculated a positive, neutral, and negative score and ensured that the sum of the three scores equaled 1. Thus, each discussion was represented by a ratio of positive, neutral, and negative sentiments. The sentiment with the most dominant score was then labeled.

We evaluated trends in sentiments over time by using the Mann–Kendall test, which assesses whether a monotonic increasing or decreasing trend occurred over time. We also analyzed the trends by drug, evaluated the score within each label (e.g., negative scores for negative labels), and the yearly proportion of topics within each cluster.

### Statistical analysis

All statistical data were analyzed using Python, version 3.8.8 (Python Software Foundation), alongside several essential libraries, including scikit-learn, version 1.3.1; BERTopic, version 0.15.0; transformers, version 4.33.2; and matplotlib, version 3.7.3. The characteristics of the posts and comments were quantitatively described using mean values and standard deviations. Trends were analyzed through the original Mann–Kendall tests using the PyMannKendall package, version 1.4.3. A p-value of 0.05 was considered statistically significant. To enhance the statistical power of the analyses, we used annual mean values. Codes are available on https://github.com/ABMI/reddit-psychiatry/tree/main.

## Results

**Fig 2:**
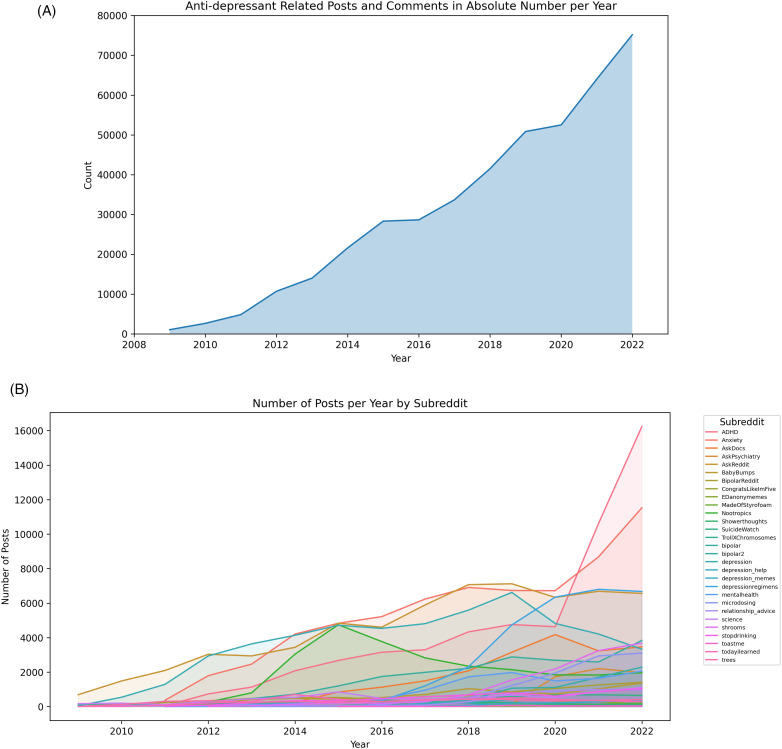
Antidepressant related posts and comments over time. (A) the annual count of posts and comments related to antidepressants from 2010 to 2022.The shaded area represents the cumulative number of posts and comments. (B) Frequency of antidepressant-related posts/comments over time by subreddit.

AI driven topic clustering and trend analysis workflow for this study is depicted in (Fig 1, Fig 2).

After searching for “depression” and “antidepressant” within the platform’s search engine, we identified 29 subreddits relevant to both queries. In 819,739 discussions, subsequent topic and group clustering, excluding off-topic discussions, resulted in a refined dataset of 429,510 discussions (Table 1). Within the generic and brand names of specific antidepressants, the largest single-word search terms were selective serotonin reuptake inhibitors (SSRIs), including “Lexapro”, “zoloft,” and “prozac”, which accounted for 13.4%, 13.0%, and 10.9% of the search queries, respectively. The subreddits “Anxiety” (15.3%) and “AskReddit” (14.6%) were the most active in discussions containing prespecified keywords, followed by “ADHD” (12.5%) and “depression” (11.9%). The descriptions of the subreddits included are provided in S2 Table.Furthermore, descriptive analysis identified 204,035 unique authors.

**Table 1:** Discussion Summary Statistics.

Characteristic	All discussions	Comments	Posts
**Discussion Metrics**			
Total discussions, n	429,510	344,478 (80.2%)	85,032 (19.8%)
Characters, mean (SD)	512.7 (748.5)	432.3 (557.8)	838.5 (1,198.9)
Unique authors, n	204,035	169,906	52,618
**Antidepressant Mentions, n (%)**			
Lexapro (escitalopram)	57,597 (13.4%)	44,608 (12.9%)	12,989 (15.3%)
Zoloft (sertraline)	55,922 (13.0%)	45,236 (13.1%)	10,686 (12.6%)
Prozac(fluoxetine)	46,956 (10.9%)	38,049 (11.0%)	8,907 (10.5%)
Effexor (venlafaxine)	28,921 (6.7%)	23,523 (6.8%)	5,398 (6.3%)
Paxil (paroxetine)	15,132 (3.5%)	12,159 (3.5%)	2,973 (3.5%)
Cymbalta (duloxetine)	14,894 (3.5%)	11,722 (3.4%)	3,172 (3.7%)
**Subreddit Distribution, n (%)**			
*Mental Health-Focused*			
Anxiety	65,669 (15.3%)	49,699 (14.4%)	15,970 (18.8%)
ADHD	53,675 (12.5%)	41,698 (12.1%)	11,977 (14.1%)
Depression	51,202 (11.9%)	34,090 (9.9%)	17,112 (20.1%)
Depression Regimens	28,637 (6.7%)	24,576 (7.1%)	4,061 (4.8%)
Bipolar	20,608 (4.8%)	17,513 (5.1%)	3,095 (3.6%)
Mental Health	10,935 (2.5%)	7,216 (2.1%)	3,719 (4.4%)
Bipolar Reddit	8,352 (1.9%)	7,221 (2.1%)	1,131 (1.3%)
Bipolar 2	6,766 (1.6%)	5,895 (1.7%)	871 (1.0%)
Suicide Watch	5,050 (1.2%)	3,729 (1.1%)	1,321 (1.6%)
Depression Help	1,877 (0.4%)	1,304 (0.4%)	573 (0.7%)
*Medical/Professional Advice*			
Ask Docs	19,814 (4.6%)	11,029 (3.2%)	8,785 (10.3%)
Ask Psychiatry	6,064 (1.4%)	3,555 (1.0%)	2,509 (3.0%)
*Alternative Treatments*			
Nootropics	25,698 (6.0%)	21,760 (6.3%)	3,938 (4.6%)
Shrooms	12,206 (2.8%)	9,626 (2.8%)	2,580 (3.0%)
Microdosing	10,224 (2.4%)	8,314 (2.4%)	1,910 (2.2%)
*General Discussion*			
Ask Reddit	62,774 (14.6%)	60,825 (17.7%)	1,949 (2.3%)
Science	7,184 (1.7%)	6,982 (2.0%)	202 (0.2%)
Today I Learned	4,407 (1.0%)	4,336 (1.3%)	71 (0.1%)
*Support Communities*			
Baby Bumps	7,738 (1.8%)	6,933 (2.0%)	805 (0.9%)
Stop Drinking	6,018 (1.4%)	4,733 (1.4%)	1,285 (1.5%)
Relationship Advice	4,450 (1.0%)	4,014 (1.2%)	436 (0.5%)
Trees	4,184 (1.0%)	3,792 (1.1%)	392 (0.5%)

The volume of online discussions related to antidepressants demonstrated an increase over 14 years (Fig 2A). The frequency of antidepressant-related discussions dynamically increased in certain communities, such as “ADHD” and “Anxiety” in 2020–2022 (Fig 2B, S1 Fig 1). In addition, the annual count of antidepressant-related posts and comments increased over time after clustering (S1 Fig).

**Fig 3:**
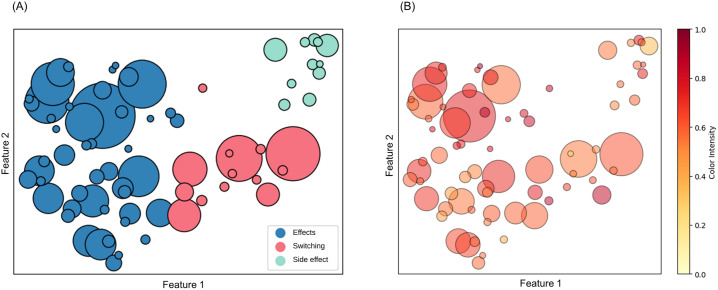
Groups of clustered topics and sentiment. (A) Representation of the clustering analysis results applied to various topics. Each cluster is denoted by a unique color; shades of blue, red, and green denote groups 1, 2, and 3, respectively. The size of each circle is proportional to the number of discussions encapsulated within that topic. The two-dimensional scatterplot positions topics according to their respective scores on Features 1 and 2, which are derived from a dimensionality reduction process using the uniform manifold approximation and projection (UMAP) technique. (B) Analysis of sentiments on clustered topics, where the color intensity corresponds to the mean negative sentiment score associated with each topic. Sentiment scores are scaled between 0 and 1, with the stronger sentiment indicated by darker hues and the weaker sentiment indicated by lighter hues.

Initially, topic clustering yielded 98 distinct antidepressant-related discussion topics from the final dataset. (S2 Fig) presents a hierarchical representation of these topics. From the 98 topics, irrelevant topics (e.g., antidepressants were only mentioned, and the topic was about something else) were excluded through a manual review by a psychiatrist, resulting in a final dataset of 73 topics (429,510 discussions). From the 73 topics categorized, three primary groups emerged according to clustering performance (Table 2, Fig 3A, S3 Fig): (1) “Benefits and side effects of antidepressants,” (2) “Drug switch, augmentation, alcohol, sertraline, escitalopram,” and (3) “Bupropion, effect, seizure, weight, augmentation.”

**Table 2:** Overview of groups of topics with example text.

Group	No. Posts	Comments	Description	Sentiment	Example text
1 ^a^	61435	240938	Benefits and side effects of antidepressants	Positive	“Cymbalta and citalopram are what worked for me in combo with amitriptyline for migraines. Antidepressants were the answer for me.”
				Negative	“I was on Paxil for about 10 years and started having the brain fog symptoms about 5-6 years being on it. it got worse so I was thinking maybe Paxil caused the brain fog”
				Negative	“started taking lexapro for my anxiety, need help I’m experiencing one of the side effects of the medicine, it makes me unable to ejaculate. is this temporary? or should I just stop taking it. I started it yesterday.”
				Negative	“recently, I’ve been reading a lot about how antidepressants are beginning to be recognized as not being effective. if this is true, biochemically speaking, why don’t they work? I have read that a series of pivotal studies determining the effectiveness of antidepressants have resulted in the conclusion that their effect is not clinically significant. it has been claimed that any benefit from taking these drugs is merely just a placebo effect.”
2 ^b^	20382	83158	Drug switch, augmentation, alcohol, sertraline, escitalopram	Negative	“Switching Zoloft to Lexapro? I’ve been on Zoloft for over 4 years now (50mg right now, but I’ve been up to 100mg) and I think it may be losing its effectiveness, as in the last year I’ve had a pretty major resurgence of symptoms that come and go.”
				Negative	“zoloft cocktail so, I’ve realized that I drink as a way to cope with my anxiety and depression. I’m on zoloft for both, and when I drink it intensifies the effects of my medication.”
				Negative	“I know the thing I really need to walk away from is the alcohol. I’ve been on a medication for it, and mixing that with therapy now (only 2 weeks in).”
				Positive	“naltrexone and lexapro helped me with my relationship with alcohol and my husband”
3 ^c^	3409	20382	Bupropion, effect, seizure, weight, augmentation	Neutral	“Does anyone have experience with bupropion? I was diagnosed with major depression, and my doctor prescribed me bupropion.
				Negative	“I took too many wellbutrin. purposely overdosed on wellbutrin 2 days ago. My stupid decision resulted in 2 seizures within a 4 hour time span.”
				Negative	“Sudden weight loss on wellbutrin? I know I have to mention this to my psychiatrist the next time I see him at the end of september, I’m just wondering if it’s common.”
				Negative	“wellbutrin and excessive sex drive ever since being on wellbutrin, I’ve had an overly excessive sex drive to the point where I become almost angry when my day lacks intimacy with my partner. has anyone else noticed this? any suggestions? this is a cross post of something I submitted at/r/sex

^a^ Topic number of Group1: 1, 4, 6, 7, 8, 9, 12, 13, 14, 15, 16, 18, 19, 24, 25, 26, 27, 30, 31, 32, 33, 36, 37, 39, 41, 44, 46, 48, 51, 53, 57, 60, 61, 62, 68, 69, 70, 74, 76, 81, 85, 86, 87, 91, 92, 93, 96, 97

^b^ Topic number of Group2: 3, 5, 10, 11, 21, 29, 59, 65, 66, 71, 77, 80, 82, 90

^c^ Topic number of Group3: 22, 23, 47, 49, 50, 55, 58, 67, 75, 78.

After reviewing the texts categorized into distinct topic clusters, we found a considerable number of discussions predominantly featuring personal experiences associated with antidepressant administration. Also, there were 5 or fewer discussions per author (S4 Fig). The first topic cluster encompasses queries about antidepressant use, with individuals sharing their personal stories, including the perceived benefits and side effects. In the second topic, the discourse shifts to experiences related to switching between different antidepressants, along with inquiries and anecdotes about the implications of consuming alcohol while on these medications. The third topic is specifically focused on bupropion, highlighting its effects and side effects, especially the risk of seizures and the potential for weight loss.

In the 429,510 online discussions, we categorized sentiments into positive, neutral, and negative. Sentiments were analyzed both by topic groups and by antidepressant types (Table 2, S1 and S3 Tables). An example of a comment with negative sentiments from Topic Cluster 1 is as follows: “I was on Paxil for about 10 years and started experiencing brain fog symptoms after 5–6 years. It worsened over time, leading me to suspect Paxil as the cause.” Conversely, a positive sentiment can be exemplified by the following comment: “Cymbalta and citalopram are what worked for me in combination with amitriptyline for migraines. Antidepressants were the answer for me.” However, negative sentiments were the most prevalent among all the sentiment categories; Fig 3B illustrates their distribution. Furthermore, the mean negative scores for each group were consistently above 0.5 (S4 Table). We also provided the mean negative sentiment score for each topic in (S5 Table).

**Fig 4:**
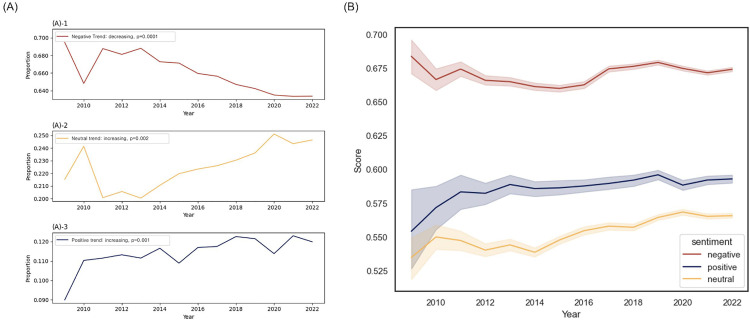
Sentiment Trends in Antidepressant. (A) Representation of proportional changes in antidepressant-related sentiments over time, showing the prevalence of negative ((A)-1), neutral ((A)-2), and positive ((A)-3) discussions. (B) Illustration of annual change in mean sentiment scores within online discussions about antidepressants.

We measured the fluctuations in sentiment proportions over time to analyze the temporal changes in sentiment trends across all the antidepressants discussed (Fig 4A, S6 Table). The negative sentiments remained to be the most frequent during the observation period, followed by the neutral sentiments and then the positive sentiments. Regarding the annual trend analysis of these sentiments over time, the proportion of negative sentiments (Mann–Kendall score: −71, z = 0.2, p = 0.0001) steadily decreased, whereas those of neutral sentiments (Mann–Kendall score: 57, z = 3.5, p = 0.0021) and positive sentiments (Mann–Kendall score: 61, z = 3.8, p = 0.0010) steadily increased (S7 Table). However, when trends were analyzed starting from 2013, no significant uptrend in positive sentiments (Mann-Kendall score: 32, z = 1.56, p = 0.1179) was observed, while the significance of trends for neutral (Mann-Kendall score:32, z = 3.23, p = 0.0012) and negative sentiments (Mann-Kendall score:-34, z = −3.44, p = 0.0006) was maintained (S8 Table).

Moreover, an additional sensitivity analysis was conducted to evaluate changes in sentiment trends over time for individual antidepressants (S9 Table). Interestingly, among all the drugs analyzed for trend, SSRIs and serotonin and norepinephrine reuptake inhibitors (SNRIs) showed a significant decline in negative sentiments alongside an increase in neutral sentiments. Overall, for each drug, negative sentiments exhibited either a stable pattern or a downward trend, whereas neutral and positive sentiments displayed either a stable pattern or an upward trend. No significant upward trends in negative sentiments or downward trends in positive or neutral sentiments were observed.

Next, we analyzed the change in the strength of the probability of classifying sentiments into their designated labels over time (Fig 4B). For instance, we examined the trend of how dominant the proportion of negatives was within the group classified as negative over the years. The probability of sentiment classification within the neutral sentiments (Mann–Kendall score: 65, z = 3.5, p = 0.0005) and positive sentiments (Mann–Kendall score: 71, z = 3.8, p = 0.0001) also showed significant rise (S10 Table).

To better understand the observed trends, we conducted a further analysis of the annual changes in topic proportions within each cluster, focusing specifically on topics that constituted at least 3% of the discussions within the cluster per year (Table 3). Then, two example text from given topic is randomly selected and provided in (S11 Table). In cluster 1, we observed a notable increase in the proportion of discussions (Mann-Kendall score: 75, z = 4.05, p = 0.0001) related to ADHD (topic 1), involving terms such as “ADHD,” “ADHD medication,” and “antidepressant effect” A case from the discussions illustrates patient’s inquiry about the use of antidepressant and ADHD medication together. Concurrently, there was a decrease in discussions concerning the side effects of antidepressants, including Prozac (Mann-Kendall score: −71, z:-3.8, p = 0.0001, topic 4), sexual side effects (Mann-Kendall score:-41, z:-2.19, p = 0.0285, topic 7), and Effexor (Mann-Kendall score: −69, z:-3.72, 0.0002, topic 8). In cluster 2, a decline in Topic 5(Man-Kendall score:-79, z:-4.27, p = 0.0002) is observed, which is characterized by terms such as “stop,” “dose,” “side effect,” and “withdrawal from Zoloft.”. An illustrative example from this topic is: “I stopped taking my zoloft for a solid week. I had a constant headache and cried all the time.” Conversely, Topic 10, which includes terms like “switch,” “augmentation,” and “stop from sertraline,” exhibited an increase (Mann-Kendall score:87, z: 4.71, p = 0.00003). For cluster 3, there was an increase in discussions pertaining to the benefits of bupropion (Mann-Kendall score: 57, z: 3.06, p = 0.0022, topic 22) A representative example from these discussions is: “I’m very happy you found meds that work. (bupropion).” In contrast, the seizure related topics with bupropion decreased (Mann-Kendall score: −59, z: −3.18, p = 0.0015, topic 55).

**Table 3:** Annual Trends for Topic Proportions Related to Antidepressant Discussions Across Three Clusters.

Cluster	Topic descriptionᵇ		Trend	p-value	z	Tau	s	Slope	Int.ᵃ
1	1	ADHD, ADHD medications, antidepressants effect, misdiagnosis	increasing	0.000051	4.051	0.824	75	1.272	4.643
	4	Prozac side effects	decreasing	0.000127	−3.832	−0.78	−71	−0.613	13.67
	6	SSRI side effects	no trend	0.51122	−0.657	−0.143	−13	−0.131	8.36
	7	Sexual side effects	decreasing	0.028539	−2.19	−0.451	−41	−0.164	8.289
	8	Effexor side effects	decreasing	0.000197	−3.723	−0.758	−69	−0.356	9.77
2	3	Treatment switching, augmentation, and discontinuation with Lexapro	no trend	0.15463	−1.423	−0.297	−27	−0.244	35.368
	5	Treatment discontinuation, dosing, and adverse effects with Zoloft	decreasing	0.000195	−4.27	−0.868	−79	−0.76	29.791
	10	Treatment switching, augmentation, and discontinuation with sertraline	increasing	0.000025	4.708	0.956	87	1.063	3.464
	11	Augmentation of trazodone, insomnia	no trend	0.188887	1.314	0.275	25	0.147	10.439
	21	Alcohol drinking, augmentation with alcohol and antidepressants	decreasing	0.048745	−1.971	−0.407	−37	−0.182	7.589
3	22	Therapeutic benefits and medication switching with bupropion	increasing	0.002172	3.066	0.626	57	0.842	16.303
	23	Therapeutic benefits and recommendations for Wellbutrin as an antidepressant	no trend	0.22844	1.204	0.253	23	0.184	22.314
	47	Side effects, switching from bupropion, no benefit from both Lexapro and Wellbutrin	no trend	0.82666	0.219	0.055	5	0.009	7.891
	49	Seizure risk of bupropion	decreasing	0.048745	−1.971	−0.407	−37	−0.366	9.97
	55	Adding bupropion to Zoloft, adding Zoloft to bupropion	decreasing	0.001497	−3.175	−0.648	−59	−0.301	8.949
	58	Bupropion for depression, No effects	no trend	0.381074	−0.876	−0.187	−17	−0.081	6.019

ᵃInt: Intercept, ᵇSSRI: Selective Serotonin Reuptake Inhibitor, ᶜDrug names in topic descriptions reflect the original search terms. Brand name medications and their generic equivalents are: Lexapro (escitalopram), Zoloft (sertraline), Prozac (fluoxetine), Effexor (venlafaxine), Wellbutrin (bupropion), Prozac (fluoxetine), Effexor (venlafaxine), Lexapro (escitalopram), Zoloft (sertraline), Wellbutrin (bupropion)

Lastly, the yearly change in sentiment proportion within represented topics in (S5 Fig) to show the annual sentiment composition difference among the topics and proportional change of each sentiment within the topic.

## Discussion

Our study examined 429,510 online discussions from at most 204,035 unique authors across various subreddits from 2009 to 2022, assuming no duplicate accounts. This dataset, obtained from an initial pool of over 800,000 discussions, provides both public perception and individual experiences with antidepressant use, providing a unique perspective on real-world concerns and sentiments toward antidepressants.

Among the discussions about antidepressants over 14 years, medications such as Lexapro and Zoloft were most frequently mentioned, aligning with their prevalent use in clinical settings. The subreddit “Anxiety” appeared as one of the leading platforms for these discussions, suggesting that concerns and inquiries related to antidepressants are highly frequent within communities engaged in anxiety-related topics.

Through topic modeling, we identified 73 distinct discussion themes. The topics were grouped into (1) the benefits and side effects of antidepressants, (2) drug switching and the interaction with alcohol, and (3) specific discussions around bupropion. This categorization underlines the community’s interest in realistic, experience-based information beyond what is typically available through a clinical context.

The cluster 1, which encompasses the largest number of topics compared to other clusters, primarily addresses the benefits and side effects associated with antidepressants, indicating the major concern for antidepressants among patients. Topics related to side effects or specific circumstances include changes in sex drive (topic 7), pregnancy (topic 18), nausea (topic 37,97), weight gain (topic 27), brain zaps, fog (topic 44, 96), memory loss (topic 51), No effect in anhedonia (topic 60), hair loss (topic 92), and diarrhea (topic 93) These aspects are likely subjects of interest among individuals and may require comprehensive explanation within clinical settings.

While cluster 1 primarily focused on the side effects and benefits of antidepressants, cluster 2 addressed concerns for switching, augmenting, or discontinuing antidepressants. Among the topics in the cluster, particularly extracted medications as a topic keyword were sertraline (Zoloft), escitalopram (Lexapro), and trazodone (for insomnia). This may indicate that patients may have many worries or concerns whether to switch, augment or stop from these medications. In addition, we found that a patient’s significant focus was on medication interactions, particularly with alcohol. Patients may not be informed about the interaction of alcohol consumption with antidepressants, or they may hesitate to ask their physicians about alcohol intake because of anticipated negative responses from healthcare providers, [30] indicating a barrier to open communication.

Cluster 3 addressed issues with the use of bupropion, a medication categorized as a norepinephrine and dopamine reuptake inhibitor (NDRI). It finds application not only in smoking cessation but also off-label for conditions such as ADHD [31] and weight loss [32]. This unique classification, owing to its diverse therapeutic utility compared to conventional antidepressants, implies its categorization into a distinct cluster.

The sentiment analysis further enriches our understanding of patient perception. Negative sentiments were the most predominant among the three sentiments, indicating its possible link with medication adherence, considering that the clustered groups contained topics about the antidepressants’ side effects and drug switching. Therefore, the importance of in-depth explanations about the drugs’ effects and potential side effects should be further emphasized [33].

The sentiment trends related to antidepressant use have also revealed the public perception of these medications over time. The consistent predominance of negative sentiments throughout the observation period highlights the challenges and concerns associated with antidepressant use. However, our annual trend analysis showed a gradual shift, with a steady decrease in negative sentiments and an increase in neutral sentiments. This trend was also consistent in the sensitivity analyses of SSRIs and SNRIs. This shift may reflect greater acceptance and understanding of mental health challenges and the role of medication in managing these conditions [34]. It may also indicate improved experiences with antidepressants, perhaps because of better patient education, more effective treatment approaches, or advancements in the development of these drugs.

Furthermore, the observed increase in the certainty of sentiment classification over time, especially within the neutral and positive sentiment categories, suggests that public opinions on antidepressants are becoming more pronounced and definitive. This trend may reflect a growing body of shared experiences and knowledge among users, fostering a community of informed individuals who can confidently articulate their perspectives.

To further elucidate the observed shift in sentiment, additional analyses were conducted to assess changes in the annual proportions of major topics within clusters. In cluster 1, there has been an increasing trend in the discussion of topics related to ADHD and antidepressants, encompassing terms such as ‘ADHD,’ ‘ADHD medications’ and ‘antidepressants effects’. This trend may align with previous studies indicating an increase in ADHD diagnoses, and findings suggesting that a concomitant diagnosis of ADHD with depression is prevalent [35]. Moreover, while discussions about the side effects of antidepressants have diminished in Cluster 1 (topics 4, 7, 8), Cluster 2 (topic 5), and Cluster 3 (topic 55), there has been an increase in discussions on the benefits of bupropion within Cluster 3 (topic 22).This shift could indicate that patients are becoming more aware of the effects and side effects of antidepressants as their use accumulates over time, which may correspond with a decrease in negative sentiment and an increase in neutral sentiment.

Research into public perceptions of antidepressants has evolved alongside data collection methodologies. Earlier studies often relied on survey data to gauge attitudes. [36–38] More recent investigations have leveraged diverse sources, including social media platforms, to understand contemporary viewpoints [39,40]. However, a significant gap remains in understanding the longitudinal shifts in these perceptions. While the majority of existing research offers cross-sectional snapshots, few studies have examined how public opinion has changed over time. Notably, a study conducted in 2011 provided a valuable trend analysis [36], but the landscape of online discourse and public sentiment has undoubtedly shifted considerably since then. Our current study offers a follow-up, providing results aligning with previous findings and insights into the evolving public perception of antidepressants as reflected in longitudinal Reddit data.

### Limitations

Although this study offers significant findings on public perceptions and trends in sentiment change, its inherent limitations should also be acknowledged. First, the data represent self-selected individuals willing to share their experiences on a public platform; thus, such data may not fully represent the broader population. Additionally, the inherent anonymity of users on the employed social media platform constrained us from identifying the demographic characteristics of individuals who posted and commented on the platform. Furthermore, social media user demographics are generally skewed toward a younger population, mostly ranging from 18 to 29 years; [16] While this is a population that was less discussed in previous studies and can be harder to reach with traditional research based on medical records, this characteristic could have influenced the nature and context of the discussions analyzed. Additionally, rather than drawing the analysis from all posts and comments found on Reddit, we narrowed the scope to subreddits dedicated to discussions about antidepressants. Consequently, this approach may have excluded relevant posts and comments from other areas of the social media platform not encompassed within these specified communities.

The geographic distribution of the data is also uncertain. While Reddit is mostly based on English, and started out as a platform based on English-speaking countries, the nature of online platforms makes it difficult to accurately pinpoint the physical distribution of the target audience.

Other important limitations worthy of discussion are the potential existence of automated “bot” accounts, and the potential involvement of industry in the community. Since Reddit allows bot activities, the possibility of pharmaceutical companies running such bots in order to drive the general discussion towards how they intend the public perception to be cannot be immediately ruled out. However, it is also worth noting that the data used in this study was collected until 2022, before generative AI models such as ChatGPT went into service. Therefore it was much easier to notice when certain accounts were posting automated sentences. Furthermore, the amount of data analyzed in this study makes it hard for certain entities to change the direction of the sentiment entirely.

Lastly, spelling inaccuracies may result in the erroneous categorization of discussions, thereby attributing a false-positive sentiment related to antidepressant use.

## Conclusions

In summary, the negative perceptions of antidepressants remain high on social media, with topics about drug effectiveness and side effects being the dominant topics. However, negative sentiments gradually decreased, while neutral sentiments increased over 10 years. This shift may align with a decrease in yearly proportion of side effect related topics within each cluster and an increase in discussions highlighting the benefits of antidepressants, particularly bupropion. While the true cause of the change in trend can include multiple factors such as market involvement or endeavor in patient education, these trends and information may help improve strategies to address barriers to antidepressant use and adherence.

## Supporting information

S1 Fig Antidepressant related post and comment over time. (DOCX)

S1 Table List of antidepressant keywords collected in this study. (DOCX)

## List of abbreviations:

ADHD: Attention Deficit Hyperactivity Disorder
API: Application Programming Interface
BERT: Bidirectional Encoder Representations from Transformers
c-TF-IDF: Class-based Term Frequency Inverse Document Frequency
MDD: Major Depressive Disorder
NDRI: Norepinephrine and Dopamine Reuptake Inhibitor
NLP: Natural Language Processing
SNRIs: Serotonin and Norepinephrine Reuptake Inhibitors
SSRIs: Selective Serotonin Reuptake Inhibitors
UMAP: Uniform Manifold Approximation and Projection
