# Supplementary material for: Public perception and changing attitudes toward antidepressants over a decade in social media: Lessons learned from online discussion using artificial intelligence
Source: PLoS One. 2025 Sep 4;20(9):e0318464. doi: 10.1371/journal.pone.0318464 (PMC12410866; doi:10.1371/journal.pone.0318464)
Supplement: S1 Fig — (DOCX) [file pone.0318464.s001.docx]

**Supplementary Information(Figures)**

**Contents**

**Supplementary Figure 1. Antidepressant related post and comment over time**

**Supplementary Figure 2. Hierarchical representations of the extracted topics**

**Supplementary Figure 3. Group Determination Sensitivity Analysis.**

**Supplementary Figure 4. Histogram of Number of Discussions Per Author**

**Supplementary Figure 5. Yearly change in sentiment proportion within represented topics.**

(A)


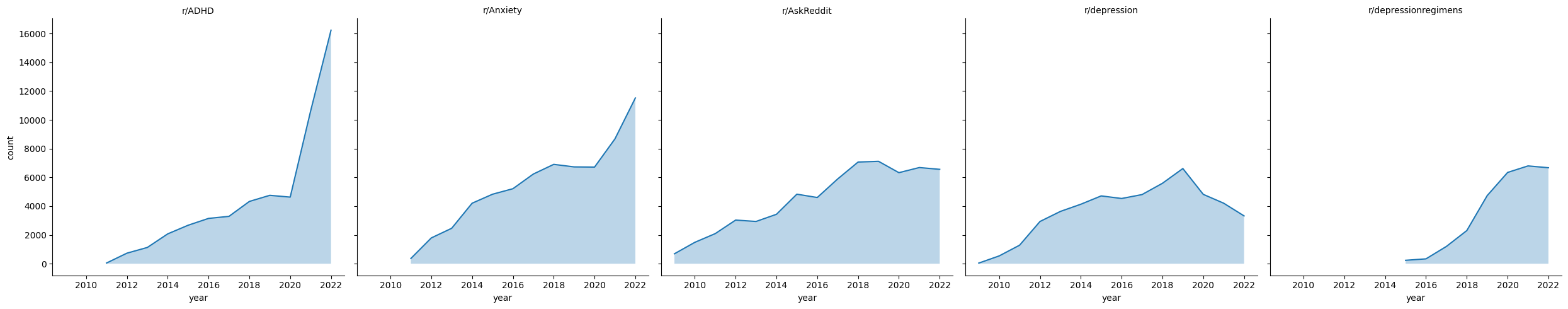

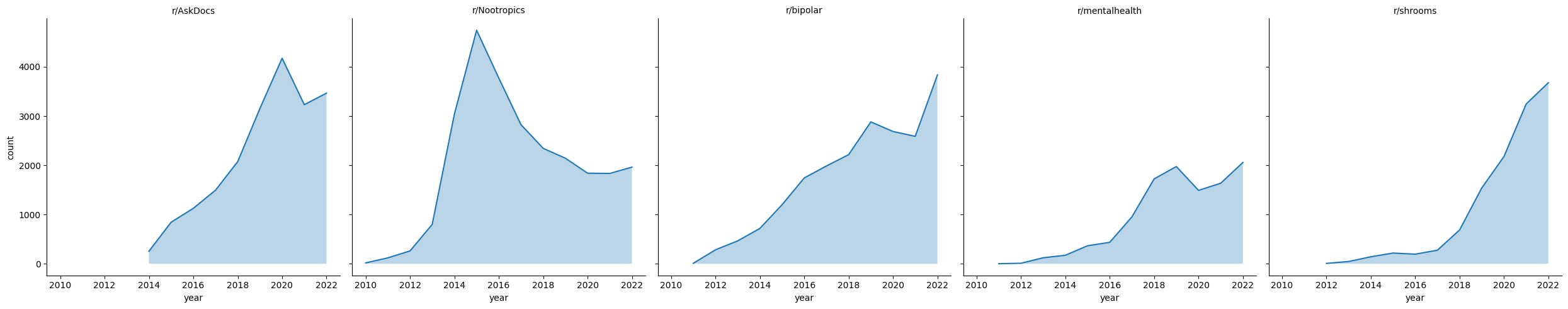

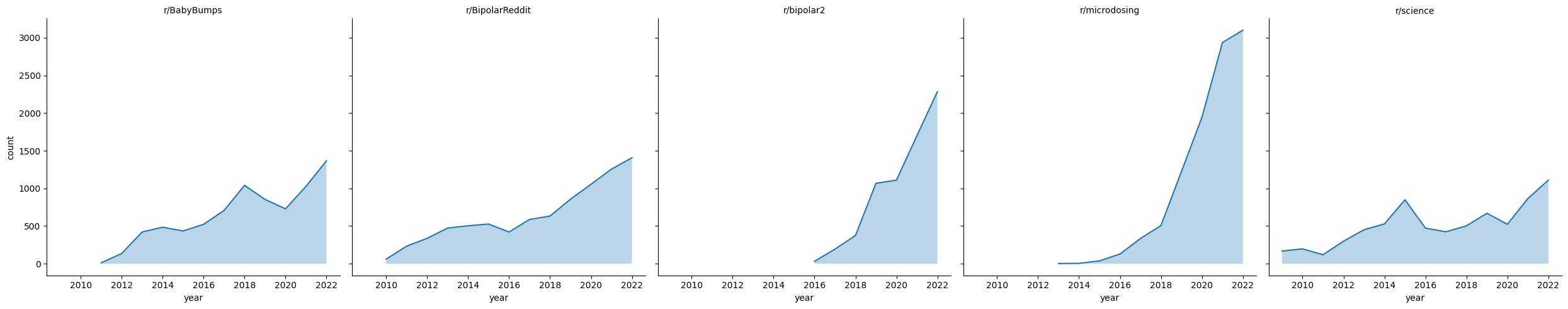

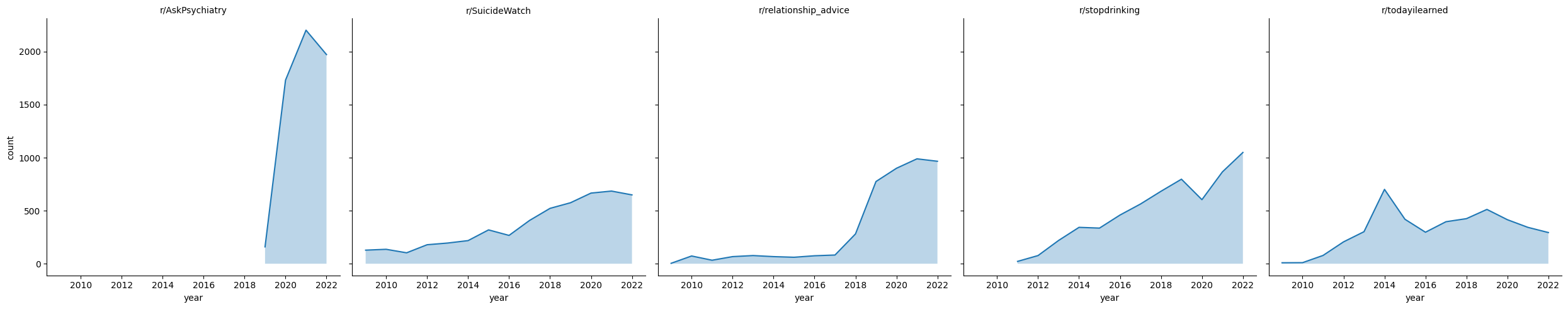

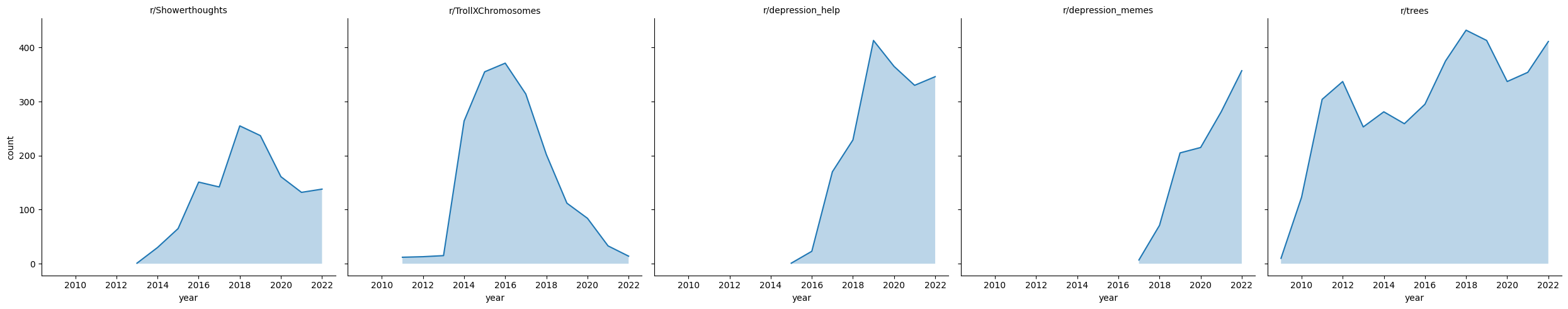

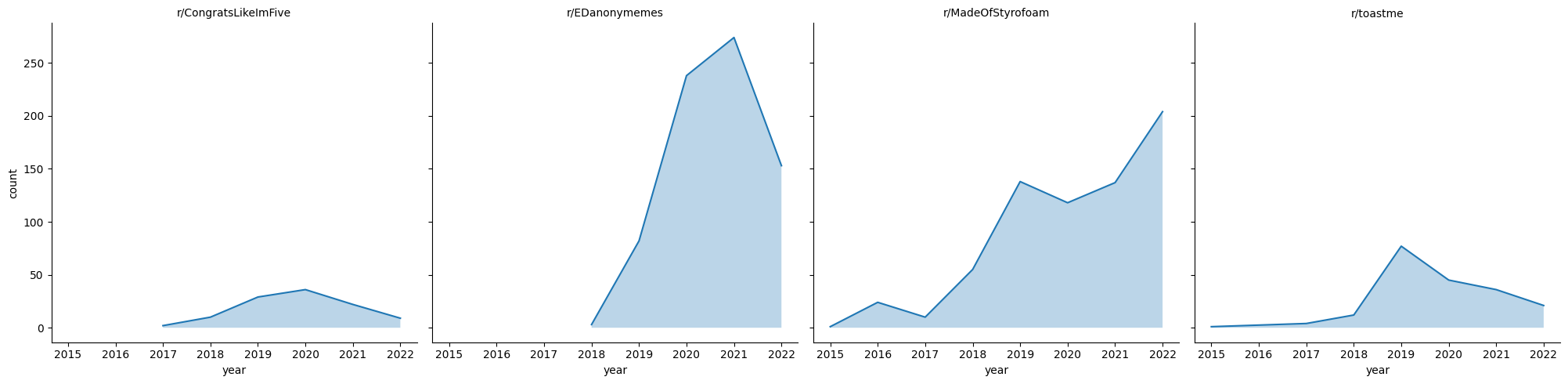


(B)


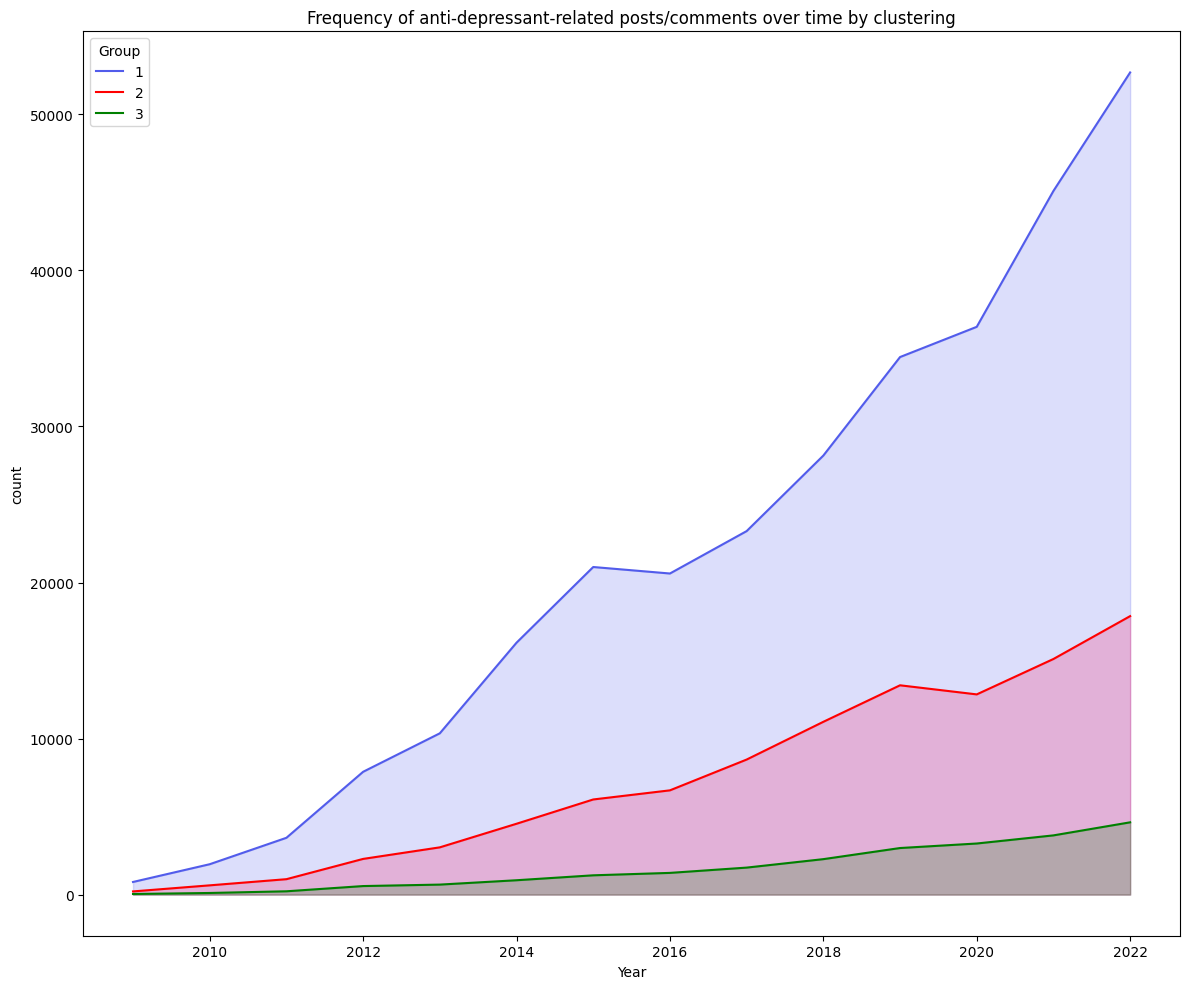


**Supplementary Figure 1. Antidepressant related post and comment over time**

(A) number of all posts and comments for each subreddit over time (B) frequency of anti-depressant-related post/comment over time by clustering.
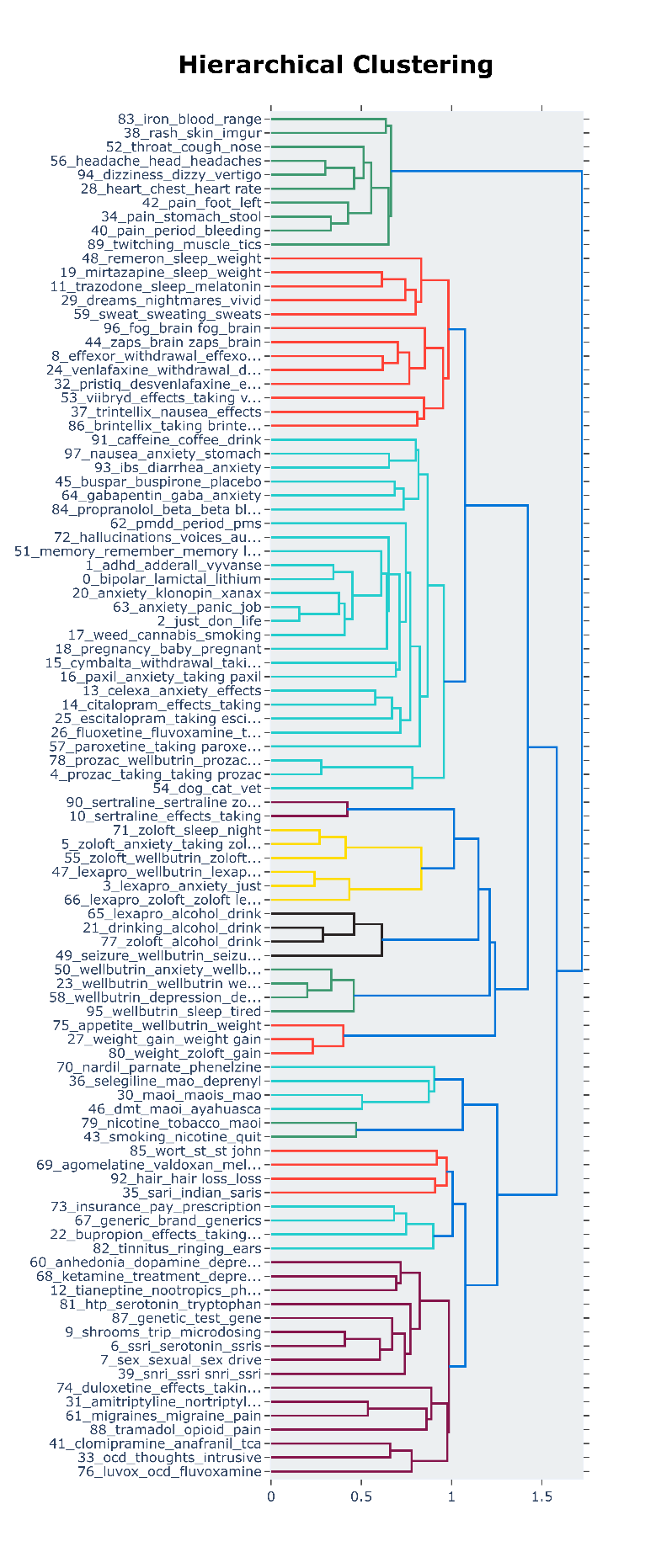


**Supplementary Figure 2. Hierarchical representations of the extracted topics**

The y-axis represents the depth of the hierarchical tree corresponding to each node in the dendrogram. Each number denotes a specific topic, followed by the associated keywords


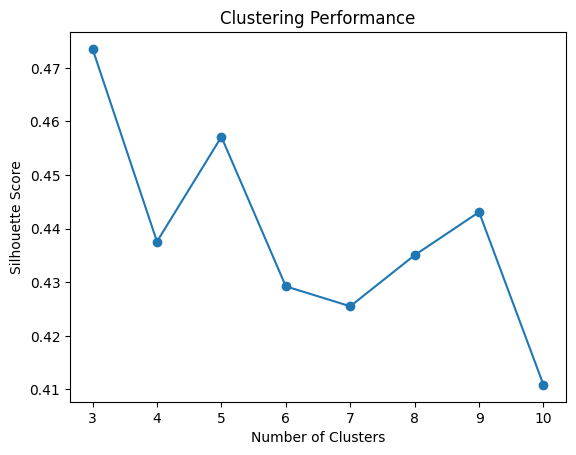


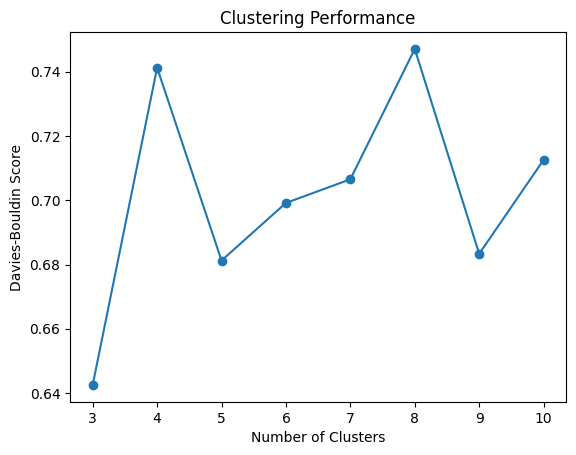


**Supplementary Figure 3. Group Determination Sensitivity Analysis**

Optimization of the number of groups for aggregating the 73 topics was evaluated using the Silhouette score (top) and Davies-Bouldin score (bottom). Optimal clustering performance is indicated by a higher Silhouette score and a lower Davies-Bouldin score, both of which reached their peak at n = 3 groups.


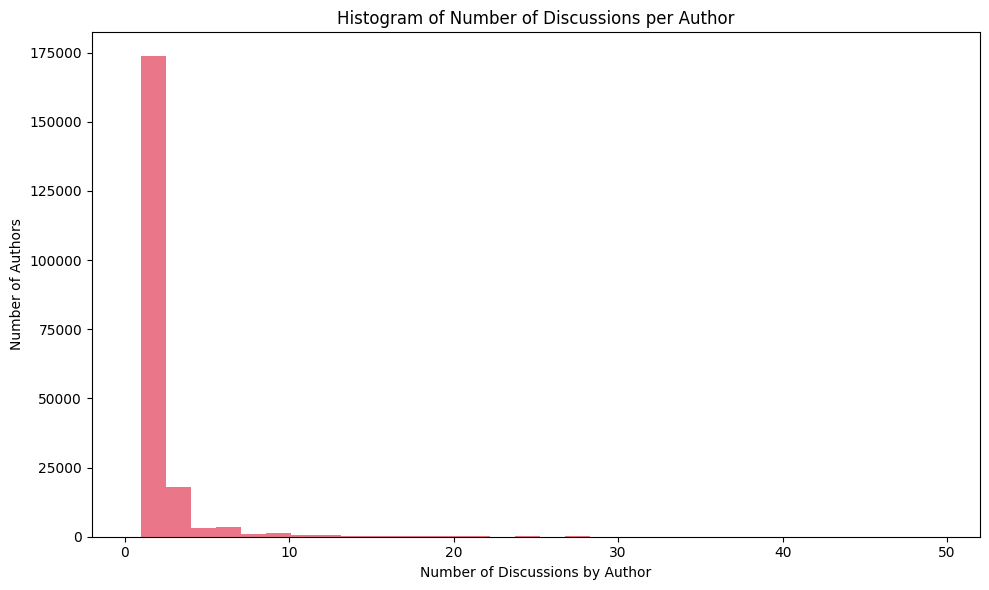


**Supplementary Figure 4. Histogram of Number of Discussions Per Author**

This figure illustrates the number of discussions contributed by each author.


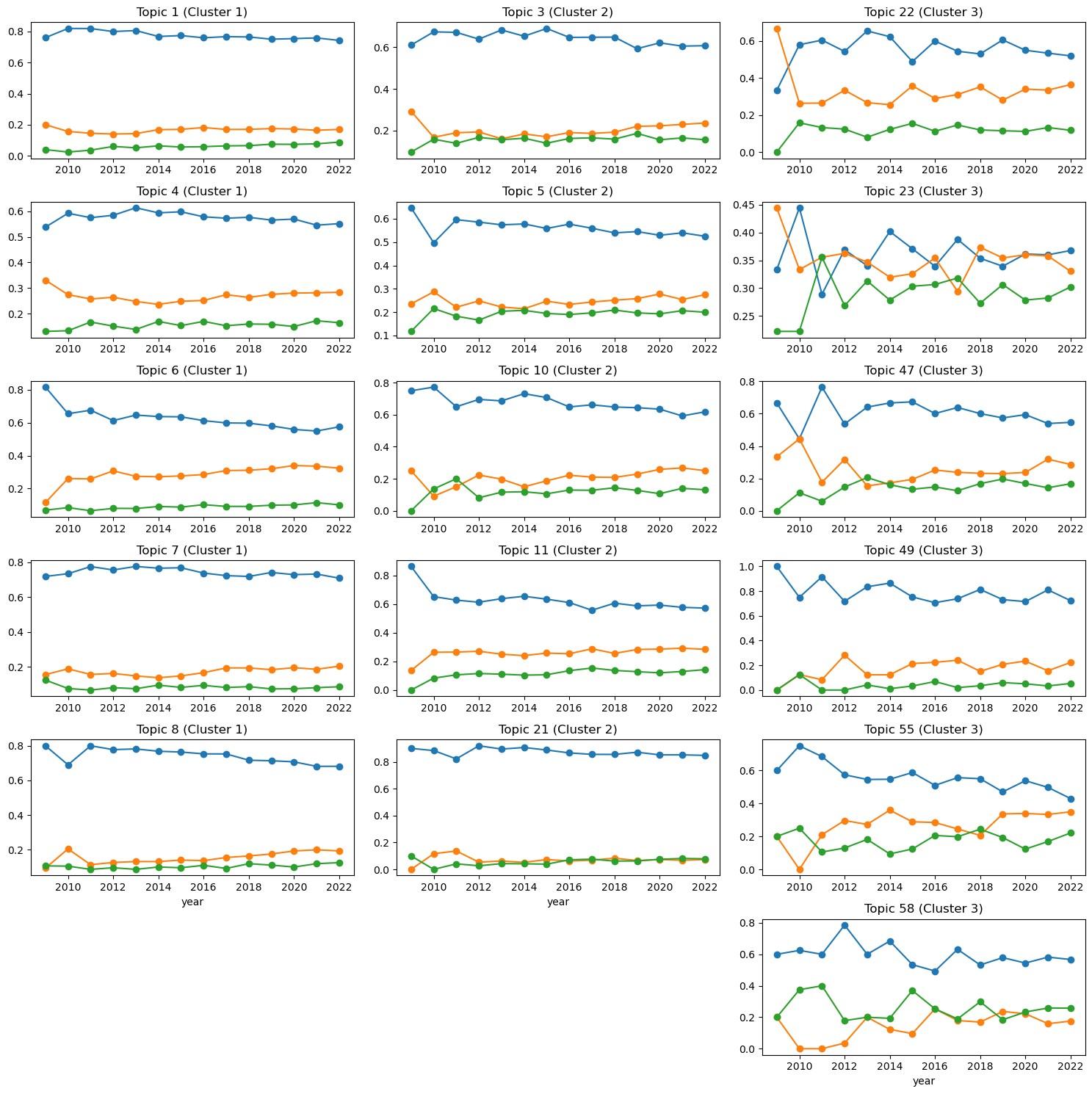


**Supplementary Figure 5. Yearly change in sentiment proportion within represented topics**

Each sentiment is illustrated with corresponding color: Negative sentiment(blue), Neutral sentiment(orange), Positive sentiment(green)
