# Supplementary material for: Public perception and changing attitudes toward antidepressants over a decade in social media: Lessons learned from online discussion using artificial intelligence
Source: PLoS One. 2025 Sep 4;20(9):e0318464. doi: 10.1371/journal.pone.0318464 (PMC12410866; doi:10.1371/journal.pone.0318464)
Supplement: S1 Table — (DOCX) [file pone.0318464.s002.docx]

**Supplementary Information(Tables)**

**Contents**

**Supplementary Table 1. List of antidepressant keywords collected in this study.**

**Supplementary Table 2. Description of the subreddits included**

**Supplementary Table 3. Type of antidepressants and related topics**

**Supplementary Table 4. Proportion of negative Sentiment Across Groups**

**Supplementary Table 5. Mean Sentiment Across Topics**

**Supplementary Table 6. Annual mean sentiment scores**

**Supplementary Table 7. Annual trend test using Mann Kendall method for total drugs.**

**Supplementary Table 8. Annual trend test using Mann Kendall method for total drugs starting from 2013.**

**Supplementary Table 9. Annual trend test using Mann Kendall method for each drug and class.**

**Supplementary Table 10. Annual trend test for strength of the probability in classifying sentiments into their designated categories using Mann Kendall method for total drugs.**

**Supplementary Table 11. Summary and examples of discussion topics related to Antidepressant use illustrated in figure 3**

| **Class** | **Generic name (brand name)** |
| --- | --- |
| **selective serotonin reuptake inhibitors (SSRIs)** | sertraline (Zoloft) |
|  | fluoxetine (Prozac, Sarafem, Symbyax) |
|  | citalopram (Celexa) |
|  | escitalopram (Lexapro) |
|  | paroxetine (Paxil, Pexeva, Brisdelle) |
|  | fluvoxamine (Luvox) |
| **Serotonin partial agonist/reuptake inhibitors (SPARIs)** | Vilazodone (VIIBRYD) |
| **Serotonin-norepinephrine reuptake inhibitors (SNRIs)** | desvenlafaxine (Pristiq, Khedezla) |
|  | duloxetine (Cymbalta, Drizalma, Irenka) |
|  | levomilnacipran (Fetzima) |
|  | Milnacipran (Savella). |
|  | venlafaxine (Effexor) |
| **Selective norepinephrine reuptake inhibitors (NRIs)** | Agomelatine (Valdoxan) |
| **Norepinephrine–dopamine reuptake inhibitors (NDRIs)** | Bupropion (Wellbutrin, Forfivo, Aplenzin), |
| **Serotonin antagonist/reuptake inhibitors (SARIs)** | nefazodone (Serzone) and trazodone (Desyrel, Desyrel Dividose, Oleptro, Trazodone) |
| **Noradrenergic and specific serotonergic antidepressant (NaSSA)** | Mirtazapine (Remeron) |
| **TCA** | amitriptyline (Elavil) |
|  | amoxapine (Asendin) |
|  | clomipramine (Anafranil) |
|  | desipramine (Norpramin) |
|  | doxepin (Silenor) |
|  | Dothiepin (prothiaden) |
|  | Lofepramine (deprimyl) |
|  | Tianeptine (stablon, coaxil) |
|  | Maprotiline (ludiomil) |
|  | imipramine (Tofranil) |
|  | nortriptyline (Pamelor) |
|  | protriptyline (Vivactil) |
|  | trimipramine (Surmontil) |
| **MAOi** | isocarboxazid (Marplan) |
|  | phenelzine (Nardil) |
|  | selegiline (Emsam)[transdermal patch] |
|  | tranylcypromine (Parnate) |
| **Others** | vortioxetine (Brintellix, Trintellix) |

**Supplementary Table 1. list of antidepressant keyword collected in this study**

| **Subreddit** | **Description** |
| --- | --- |
| r/ADHD | A community for those affected by ADHD to share experiences and strategies. |
| r/Anxiety | A supportive space for individuals dealing with anxiety disorders. |
| r/AskDocs | A subreddit where verified medical professionals answer health-related questions. |
| r/AskPsychiatry | A forum for discussing psychiatric topics with professionals and peers. |
| r/AskReddit | A place to ask and answer thought-provoking questions. |
| r/BabyBumps | A community for pregnant individuals to share experiences and seek advice. |
| r/BipolarReddit | A subreddit for discussions and support related to bipolar disorder. |
| r/CongratsLikeImFive | A place to share achievements and receive positive reinforcement. |
| r/EDanonymemes | A community for sharing memes related to eating disorders. |
| r/MadeOfStyrofoam | A subreddit for sharing and discussing surreal or absurd content. |
| r/Nootropics | A community focused on the discussion of cognitive enhancers and supplements. |
| r/Showerthoughts | A place for sharing those miniature epiphanies you have that highlight the oddities within the familiar. |
| r/SuicideWatch | A supportive community for individuals struggling with suicidal thoughts. |
| r/TrollXChromosomes | A subreddit for humorous content from a female perspective. |
| r/bipolar | A community for individuals with bipolar disorder to share experiences and support. |
| r/bipolar2 | A subreddit focused on discussions related to Bipolar II disorder. |
| r/depression | A supportive space for individuals dealing with depression. |
| r/depression_help | A community offering support and advice for those struggling with depression. |
| r/depression_memes | A place to share memes related to depression, aiming to provide humor and relatability. |
| r/depressionregimens | A subreddit discussing routines and strategies to manage depression. |
| r/mentalhealth | A community dedicated to discussions and support for various mental health issues. |
| r/microdosing | A subreddit focused on the practice of microdosing psychedelics for mental health and cognitive benefits. |
| r/relationship_advice | A place to seek and offer advice on relationships. |
| r/science | A community for discussing science topics and sharing scientific research. |
| r/shrooms | A subreddit dedicated to discussions about psychedelic mushrooms. |
| r/stopdrinking | A support community for individuals who are trying to quit drinking alcohol. |
| r/toastme | A subreddit where users post pictures of themselves to receive compliments and positive comments. |
| r/todayilearned | A place to share interesting facts that you have learned recently. |
| r/trees | A community for cannabis enthusiasts to share experiences and information. |

**Supplementary Table 2. Description of the subreddits included**

| **Types of antidepressants** | **Topics** |
| --- | --- |
| SSRI | 3,4,5,6,10,13,14,16,18,25,26,39,47,53,57,65,66,71,76,77,78,80,81,90,96 |
| Escitalopram | 3,25,47,65,66 |
| Citalopram | 14 |
| Fluoxetine | 4,26,78 |
| Sertraline | 5,10,18,66,71,77,80,90 |
| Paroxetine | 16,57 |
| Fluvoxamine | 76 |
| SNRI | 8,15,24,32,39,74 |
| Venlafaxine | 8,24 |
| Desvenlafaxine | 32 |
| Duloxetine | 15,74 |
| NDRI (Bupropion) | 1,22,23,47,49,50,58,67,75,78,82,95 |
| NaSSA (Mirtazapine) | 19 |
| SARI (Trazodone) | 11 |
| TCA | 12,31,41,61,93 |
| MAO inhibitor | 30,36,46,70 |
| Others (Agomelatine) | 69 |
| Others (Brintellix) | 37,86 |
| Others (Ketamine) | 68 |

**Supplementary Table 3. Type of antidepressants and related topics**

| Group | Proportion of negative Sentiment |
| --- | --- |
| 1 | 0.65 |
| 2 | 0.64 |
| 3 | 0.58 |

**Supplementary Table 4. Proportion of negative Sentiment Across Groups**

Mean of the average sentiment score for all discussions by group. The value represents the proportion of negative sentiment within the overall sentiment. For example, if it is 0.7, it means that 70% of the entire content is characterized as negative sentiment.

| Group | Mean Sentiment | Group | Mean Sentiment | Group | Mean Sentiment |
| --- | --- | --- | --- | --- | --- |
| 0 | 0.75 | 46 | 0.45 | 92 | 0.79 |
| 1 | 0.76 | 47 | 0.59 | 93 | 0.72 |
| 2 | 0.94 | 48 | 0.55 | 94 | 0.99 |
| 3 | 0.63 | 49 | 0.76 | 95 | 0.64 |
| 4 | 0.57 | 50 | 0.74 | 96 | 0.76 |
| 5 | 0.55 | 51 | 0.88 | 97 | 0.93 |
| 6 | 0.59 | 52 | 0.98 |  |  |
| 7 | 0.73 | 53 | 0.61 |  |  |
| 8 | 0.73 | 54 | 0.73 |  |  |
| 9 | 0.68 | 55 | 0.51 |  |  |
| 10 | 0.64 | 56 | 0.97 |  |  |
| 11 | 0.59 | 57 | 0.64 |  |  |
| 12 | 0.56 | 58 | 0.57 |  |  |
| 13 | 0.65 | 59 | 0.87 |  |  |
| 14 | 0.67 | 60 | 0.73 |  |  |
| 15 | 0.69 | 61 | 0.77 |  |  |
| 16 | 0.72 | 62 | 0.73 |  |  |
| 17 | 0.79 | 63 | 0.99 |  |  |
| 18 | 0.71 | 64 | 0.70 |  |  |
| 19 | 0.59 | 65 | 0.56 |  |  |
| 20 | 0.75 | 66 | 0.51 |  |  |
| 21 | 0.86 | 67 | 0.51 |  |  |
| 22 | 0.55 | 68 | 0.77 |  |  |
| 23 | 0.36 | 69 | 0.40 |  |  |
| 24 | 0.67 | 70 | 0.55 |  |  |
| 25 | 0.61 | 71 | 0.67 |  |  |
| 26 | 0.58 | 72 | 0.89 |  |  |
| 27 | 0.75 | 73 | 0.88 |  |  |
| 28 | 0.95 | 74 | 0.58 |  |  |
| 29 | 0.75 | 75 | 0.58 |  |  |
| 30 | 0.56 | 76 | 0.61 |  |  |
| 31 | 0.53 | 77 | 0.64 |  |  |
| 32 | 0.60 | 78 | 0.57 |  |  |
| 33 | 0.73 | 79 | 0.56 |  |  |
| 34 | 0.97 | 80 | 0.54 |  |  |
| 35 | 0.52 | 81 | 0.60 |  |  |
| 36 | 0.47 | 82 | 0.84 |  |  |
| 37 | 0.58 | 83 | 0.79 |  |  |
| 38 | 0.95 | 84 | 0.75 |  |  |
| 39 | 0.55 | 85 | 0.61 |  |  |
| 40 | 0.94 | 86 | 0.57 |  |  |
| 41 | 0.52 | 87 | 0.69 |  |  |
| 42 | 0.98 | 88 | 0.73 |  |  |
| 43 | 0.66 | 89 | 0.92 |  |  |
| 44 | 0.84 | 90 | 0.27 |  |  |
| 45 | 0.71 | 91 | 0.79 |  |  |

**Supplementary Table 5. Mean Sentiment Across Topics**

Mean of the average sentiment score for all discussions by topic. The value represents the proportion of negative sentiment within the overall sentiment. For example, if it is 0.7, it means that 70% of the entire content is characterized as negative sentiment.

| **Year** | **Negative** | **Neutral** | **Positive** |
| --- | --- | --- | --- |
| 2009 | 0.6948 | 0.2152 | 0.0900 |
| 2010 | 0.6483 | 0.2414 | 0.1103 |
| 2011 | 0.6878 | 0.2007 | 0.1115 |
| 2012 | 0.6812 | 0.2056 | 0.1132 |
| 2013 | 0.6881 | 0.2004 | 0.1115 |
| 2014 | 0.6729 | 0.2105 | 0.1166 |
| 2015 | 0.6714 | 0.2197 | 0.1089 |
| 2016 | 0.6595 | 0.2234 | 0.1170 |
| 2017 | 0.6565 | 0.2260 | 0.1175 |
| 2018 | 0.6471 | 0.2304 | 0.1226 |
| 2019 | 0.6424 | 0.2361 | 0.1215 |
| 2020 | 0.6351 | 0.2511 | 0.1138 |
| 2021 | 0.6335 | 0.2435 | 0.1230 |
| 2022 | 0.6337 | 0.2464 | 0.1199 |

**Supplementary Table 6. Annual mean sentiment scores**

| **Sentiment** | **trend** | **h** | **p** | **z** | **Tau** | **s** | **slope** | **intercept** |
| --- | --- | --- | --- | --- | --- | --- | --- | --- |
| positive | increasing | TRUE | 0.001021 | 3.83214231 | 0.67033 | 61 | 0.001186 | 0.107463 |
| negative | decreasing | TRUE | 0.000127 | 0.218979561 | -0.78022 | -71 | -0.00524 | 0.6921 |
| neutral | increasing | TRUE | 0.002172 | 3.503672969 | 0.626374 | 57 | 0.004082 | 0.198187 |

**Supplementary Table 7: Annual trend test using Mann Kendall method for total drugs**

| **sentiment** | **trend** | **h** | **p** | **z** | **Tau** | **s** | **slope** | **intercept** |
| --- | --- | --- | --- | --- | --- | --- | --- | --- |
| positive | no trend | FALSE | 0.117850 | 1.563858 | 0.444444 | 16 | 0.000808 | 0.114222 |
| neutral | increasing | TRUE | 0.001229 | 3.231973 | 0.888888 | 32 | 0.004284 | 0.213221 |
| negative | decreasing | TRUE | 0.000580 | -3.440487 | -0.944444 | -34 | -0.005734 | 0.670022 |

**Supplementary Table 8. Annual trend test using Mann Kendall method for total drugs starting from 2013**

| **Drugs** | **sentiment** | **trend** | **h** | **p** | **z** | **Tau** | **s** | **slope** | **intercept** |
| --- | --- | --- | --- | --- | --- | --- | --- | --- | --- |
| SSRI | positive | increasing | TRUE | 0.001497 | 3.175204 | 0.648352 | 59 | 0.001991 | 0.127325 |
|  | negative | decreasing | TRUE | 0.000459 | -3.50367 | -0.71429 | -65 | -0.00761 | 0.679229 |
|  | neutral | increasing | TRUE | 0.016006 | 2.408775 | 0.494505 | 45 | 0.005298 | 0.194552 |
| Escitalopram | Positive | no trend | FALSE | 0.324424 | 0.985408 | 0.208791 | 19 | 0.001239 | 0.145528 |
|  | Negative | no trend | FALSE | 0.062698 | -1.86133 | -0.38462 | -35 | -0.00501 | 0.665868 |
|  | Neutral | no trend | FALSE | 0.062698 | 1.861326 | 0.384615 | 35 | 0.003989 | 0.181001 |
| Citalopram | Positive | increasing | TRUE | 0.003114 | 2.956224 | 0.604396 | 55 | 0.003478 | 0.098893 |
|  | Negative | decreasing | TRUE | 0.002172 | -3.06571 | -0.62637 | -57 | -0.00953 | 0.746236 |
|  | Neutral | increasing | TRUE | 0.037497 | 2.080306 | 0.428571 | 39 | 0.007192 | 0.165549 |
| Fluoxetine | Positive | increasing | TRUE | 0.000127 | 3.832142 | 0.78022 | 71 | 0.001394 | 0.578896 |
|  | Negative | no trend | FALSE | 0.826666 | 0.21898 | 0.054945 | 5 | 0.000117 | 0.671997 |
|  | Neutral | increasing | TRUE | 0.000459 | 3.503673 | 0.714286 | 65 | 0.00249 | 0.535959 |
| Sertraline | Positive | no trend | FALSE | 0.661416 | 0.437959 | 0.098901 | 9 | 0.001062 | 0.151531 |
|  | Negative | decreasing | TRUE | 0.011793 | -2.51826 | -0.51648 | -47 | -0.00614 | 0.65585 |
|  | Neutral | no trend | FALSE | 0.125311 | 1.532857 | 0.318681 | 29 | 0.003447 | 0.205125 |
| Paroxetine | Positive | increasing | TRUE | 0.008595 | 2.627755 | 0.538462 | 49 | 0.003675 | 0.07419 |
|  | Negative | decreasing | TRUE | 0.000688 | -3.39418 | -0.69231 | -63 | -0.0075 | 0.777869 |
|  | Neutral | increasing | TRUE | 0.001497 | 3.175204 | 0.648352 | 59 | 0.005016 | 0.1487 |
| Fluvoxamine | Positive | no trend | FALSE | 0.912814 | -0.10949 | -0.03297 | -3 | -0.00159 | 0.127213 |
|  | Negative | decreasing | TRUE | 0.000366 | -3.56376 | -0.72527 | -66 | -0.01975 | 0.778756 |
|  | Neutral | increasing | TRUE | 0.000824 | 3.344454 | 0.681319 | 62 | 0.025184 | 0.090015 |
| SNRI | positive | no trend | FALSE | 0.15463 | 1.423367 | 0.296703 | 27 | 0.000927 | 0.103566 |
|  | negative | decreasing | TRUE | 0.004417 | -2.84673 | -0.58242 | -53 | -0.00884 | 0.767928 |
|  | neutral | increasing | TRUE | 0.004417 | 2.846734 | 0.582418 | 53 | 0.006686 | 0.142174 |
| Venlafaxine | Positive | no trend | FALSE | 0.100518 | 1.642347 | 0.340659 | 31 | 0.00116 | 0.091069 |
|  | Negative | decreasing | TRUE | 0.001497 | -3.1752 | -0.64835 | -59 | -0.01075 | 0.808583 |
|  | Neutral | increasing | TRUE | 0.002172 | 3.065714 | 0.626374 | 57 | 0.008906 | 0.105151 |
| Desvenlafaxine | Positive | no trend | FALSE | 0.58407 | 0.547449 | 0.120879 | 11 | 0.00094 | 0.123949 |
|  | Negative | no trend | FALSE | 0.125311 | -1.53286 | -0.31868 | -29 | -0.00779 | 0.653199 |
|  | Neutral | no trend | FALSE | 0.100518 | 1.642347 | 0.340659 | 31 | 0.003082 | 0.240032 |
| Duloxetine | Positive | no trend | FALSE | 0.826666 | 0.21898 | 0.054945 | 5 | 0.002076 | 0.098922 |
|  | Negative | no trend | FALSE | 0.170475 | -1.37068 | -0.28571 | -26 | -0.00443 | 0.71966 |
|  | Neutral | no trend | FALSE | 0.188887 | 1.313877 | 0.274725 | 25 | 0.005609 | 0.161172 |
| NDRI (Bupropion) | Positive | increasing | TRUE | 0.021489 | 2.299285 | 0.472527 | 43 | 0.00183 | 0.083842 |
|  | Negative | no trend | FALSE | 0.742557 | -0.32847 | -0.07692 | -7 | -0.00091 | 0.700386 |
|  | Neutral | no trend | FALSE | 0.826666 | -0.21898 | -0.05495 | -5 | -0.00012 | 0.201999 |
| NaSSA (Mirtazapine) | Positive | no trend | FALSE | 0.324424 | 0.985408 | 0.208791 | 19 | 0.002678 | 0.098 |
|  | Negative | no trend | FALSE | 0.062698 | -1.86133 | -0.38462 | -35 | -0.00821 | 0.666167 |
|  | Neutral | increasing | TRUE | 0.048745 | 1.970816 | 0.406593 | 37 | 0.006158 | 0.23133 |
| SARI (Trazodone) | Positive | increasing | TRUE | 0.003114 | 2.956224 | 0.604396 | 55 | 0.00469 | 0.08743 |
|  | Negative | decreasing | TRUE | 0.000688 | -3.39418 | -0.69231 | -63 | -0.00731 | 0.659924 |
|  | Neutral | increasing | TRUE | 0.021489 | 2.299285 | 0.472527 | 43 | 0.003514 | 0.241849 |
| TCA | Positive | no trend | FALSE | 0.58407 | 0.547449 | 0.120879 | 11 | 0.000873 | 0.087539 |
|  | Negative | no trend | FALSE | 0.062698 | -1.86133 | -0.38462 | -35 | -0.00511 | 0.59839 |
|  | Neutral | increasing | TRUE | 0.001497 | 3.175204 | 0.648352 | 59 | 0.00496 | 0.302388 |
| MAO inhibitor | Positive | increasing | TRUE | 0.006196 | 2.737245 | 0.56044 | 51 | 0.005195 | 0.044831 |
|  | Negative | decreasing | TRUE | 0.048745 | -1.97082 | -0.40659 | -37 | -0.00615 | 0.563473 |
|  | Neutral | no trend | FALSE | 0.869352 | 0.164481 | 0.043956 | 4 | 0.000209 | 0.394989 |
| Others (Agomelatine) | Positive | no trend | FALSE | 0.271241 | 1.100209 | 0.24359 | 19 | 0.007509 | 0.079948 |
|  | Negative | no trend | FALSE | 0.854777 | 0.183027 | 0.051282 | 4 | 0.004071 | 0.407526 |
|  | Neutral | no trend | FALSE | 0.502158 | -0.6711 | -0.15385 | -12 | -0.0134 | 0.552078 |
| Others (Brintellix) | Positive | increasing | TRUE | 0.035001 | 2.108346 | 0.509091 | 28 | 0.007308 | 0.106316 |
|  | Negative | no trend | FALSE | 0.755497 | -0.3114 | -0.09091 | -5 | -0.0029 | 0.597493 |
|  | Neutral | no trend | FALSE | 0.161125 | -1.4013 | -0.34545 | -19 | -0.00673 | 0.320146 |
| Others (Ketamine) | Positive | no trend | FALSE | 0.392154 | 0.855718 | 0.192308 | 15 | 0.003372 | 0.030272 |
|  | Negative | no trend | FALSE | 0.582951 | 0.54908 | 0.128205 | 10 | 0.008691 | 0.678625 |
|  | Neutral | no trend | FALSE | 0.582951 | -0.54908 | -0.12821 | -10 | -0.00818 | 0.265287 |

**Supplementary Table 9. Annual trend test using Mann Kendall method for each drugs and class**

| **Drugs** | **sentiment** | **trend** | **h** | **p** | **z** | **Tau** | **s** | **slope** | **intercept** |
| --- | --- | --- | --- | --- | --- | --- | --- | --- | --- |
| Total | **positive** | increasing | TRUE | 0.000127032 | 3.83214231 | 0.78021978 | 71 | 0.001393847 | 0.578895993 |
|  | **negative** | no trend | FALSE | 0.82666597 | 0.218979561 | 0.054945055 | 5 | 0.000116773 | 0.671997305 |
|  | **neutral** | increasing | TRUE | 0.000458889 | 3.503672969 | 0.714285714 | 65 | 0.002490398 | 0.535959066 |

**Supplementary Table 10. Annual trend test for strength of the probability in classifying sentiments into their designated categories using Mann Kendall method for total drugs**

| **Topic/**  **Cluster** | **Model selected words** | **Topic summary** | **Two randomly selected example text** | **Negative**  **N/(%)** | **Neutral**  **N//(%)** | **Positive**  **N/(%)** |
| --- | --- | --- | --- | --- | --- | --- |
| 1/(1) | adhd, adderall, vyvanse, wellbutrin | ADHD, ADHD medications,  antidepressants effect, misdiagnosis | 1) depression and adhd meds? hi all, i just got diagnosed two weeks ago with adhd and was started on adderall, and yesteday i was diagnosed with a general mood disorder and was put on zoloft. has anyone experienced the two meds at the same time? i want some perspective on what to expect. during the two weeks of adhd meds alone i was very focused but had no motivation at all (anhedonia), and now that i'm on zoloft hopefully i'll be motivated and focused at the same time.  2) I’m on vyvanse and wellbutrin. I felt like my depression was under control | 38038 (75.72%) | 8486 (16.89%) | 3713 (7.39%) |
| 3(2) | lexapro, anxiety, just, taking | Switch, augmentation, stop from Lexapro | 1) lexapro didn't work for me. I was only taking 10mg, but i felt always on edge. i switched to an off brand celexa and that has helped.  2) as far as the lexapro goes, it will take a little time to really be effective. if you start to feel zombied, tell your psychaitrist and ask that it be changed or adjusted. | 21589 (62.8%) | 7203 (20.95%) | 5583 (16.24%) |
| 4/(1) | prozac, taking, taking prozac, just | Prozac side effects | 1) when I was 15 I was put on prozac, I ended up experiencing a lot of rage as a side effect and went off of it within a month  2) I went on prozac a few months ago. yeah, it got worse before it got better | 15513 (57.13%) | 7307 (26.91%) | 4333 (15.96%) |
| 5/(2) | zoloft, anxiety, taking zoloft, taking | Stop, dose, side effect, and withdrawal from Zoloft | 1) I stopped taking my zoloft for a solid week. i had a constant headache and cried all the time.  2) I was doing so much better and now my doctor is lowering my zoloft. it’s the end of day 2 of tapering and I got hit with a panic attack | 13546 (54.72%) | 6296 (25.44%) | 4911 (19.84%) |
| 6/(1) | ssri, serotonin, ssris, people | SSRI side effects | 1) I went on an ssri and it gave me the worst mental health week of my life  2) not sure which meds you are taking, but ssri withdrawals are horrible | 13227 (58.9%) | 7030 (31.3%) | 2200 (9.8%) |
| 7/(1) | sex, sexual, sex drive, libido | Sexual side effects | 1) I recently began to decrease the dose on prozac, mostly because i’m tired of having zero sex drive  2) I tried zoloft, but it killed my sex life | 16143 (73.46%) | 4018 (18.28%) | 1815 (8.26%) |
| 8/(1) | effexor, withdrawal, effexor xr, dose | Effexor side effects | 1) that weird wake sleep state where you can’t move is what happened to me on effexor!  2) I've been on effexor amongst other antidepressants and drank while taking them. yes, there are potential dangers but I never heard of anyone who had serious complications with it. | 14138 (72.66%) | 3226 (16.58%) | 2095 (10.77%) |
| 10/(2) | sertraline, effects, taking, taking sertraline | Switch, augmentation, stop from sertraline | 1) they took me off sertraline and put me on venlafaxine  2) I had to stop taking sertraline because it was starting to give me auras (seizure warnings). so this is really helpful for what medication to ask my doctor for next. | 8823 (63.75%) | 3254 (23.51%) | 1764 (12.74%) |
| 11/(2) | trazodone, sleep, melatonin, insomnia | Augmentation of trazodone, insomnia | 1) I'm on zoloft and also take trazodone for sleep sometimes.  2) trazodone helps me sleep otherwise no good sleeps for me. | 7610 (59.34%) | 3551 (27.69%) | 1664 (12.97%) |
| 21(2) | drinking, alcohol, drink, sober | Alcohol drinking, augmentation with alcohol and antidepressants | 1) I was very depressed, suicidal and angry in the first few weeks of sobriety. i thought it was all to much for me and started drinking.  2) I started lexapro about 5 weeks ago due to some really challenging medical issues that put me in a bad mental state. I could drink occasionally? | 5369 (86.46%) | 436 (7.02%) | 405 (6.52%) |
| 22(3) | bupropion, effects, taking bupropion, taking | Benefits from bupropion,  switch to bupropion | 1) I'm very happy you found meds that work. i tried a lot of medications before i found one that worked (bupropion).  2) I gained 40 pounds on paxil so **** that. bupropion is supposed to help some people lose weight so i’m hoping that works better for me. | 3398 (55.07%) | 2022 (32.77%) | 750 (12.16%) |
| 23(3) | wellbutrin, wellbutrin wellbutrin, taking wellbutrin, taking | Benefits of Wellbutrin,  recommending Wellbutrin as an antidepressant | 1) recently I started wellbutrin and one day i just realized i felt so relaxed.  2) I’ve only taken wellbutrin/buproprion but i remember how peaceful i felt in the first week. | 2113 (36.11%) | 2023 (34.57%) | 1716 (29.32%) |
| 47(3) | lexapro, wellbutrin, lexapro wellbutrin, wellbutrin lexapro | Side effects, switch from bupropion, no benefit from both Lexapro and Wellbutrin | 1) having a rough night dealing with lexapro/wellbutrin withdrawal. any advice or comforting words are more than welcome.  2) 300 mg of wellbutrin and 10 mg of lexapro feels like it’s doing nothing for me. | 1159 (58.83%) | 501 (25.43%) | 310 (15.74%) |
| 49(3) | seizure, wellbutrin, seizures, alcohol | Seizure risk of bupropion | 1) you need to talk to your doctor about this asap. wellbutrin increases seizure risk.  2) since wellbutrin has a seizure risk, i'd go off the wellbutrin during the cold and take pseudo-ephedrine if you can get it. | 1389 (76.11%) | 360 (19.73%) | 76 (4.16%) |
| 55(3) | zoloft, wellbutrin, zoloft wellbutrin, wellbutrin zoloft | Adding bupropion to Zoloft Adding Zoloft to bupropion | 1) I’ve had this problem with zoloft. my dr added wellbutrin to counteract it and it worked perfectly  2) I started on wellbutrin 100mg and then went up to 150, then started on zoloft 25mg for 2 weeks, 50mg for 2 weeks, 75 mg for a week, now 100mg. i don’t think starting out slow has any downsides personally. | 762 (51.42%) | 452 (30.5%) | 268 (18.08%) |
| 58(3) | wellbutrin, depression, depressed, wellbutrin depression | Bupropion for depression, No effects | 1) for what it's worth, i mentioned during my initial visit that i'd been put on wellbutrin for depression and it did nothing.  2) i tried wellbutrin. the first day i could tell a huge difference. that was immediate proof that it was a chemical imbalance &amp; not just emotional. | 749 (57.39%) | 234 (17.93%) | 322 (24.67%) |

**Supplementary Table 11. Summary and examples of discussion topics related to Antidepressant use illustrated in Figure 3. ****: improper words have been masked to ensure appropriate content presentation.**

**Standards for Reporting Qualitative Research (SRQR)**

O’Brien B.C., Harris, I.B., Beckman, T.J., Reed, D.A., & Cook, D.A. (2014). Standards for reporting qualitative research: a synthesis of recommendations. *Academic Medicine, 89(9)*, 1245-1251.

| **Pg No. Topic** | **Item** |
| --- | --- |
| **Title and abstract** |  |
| 1 Title | Concise description of the nature and topic of the study identifying the study as qualitative or indicating the approach (e.g., ethnography, grounded theory) or data collection methods (e.g., interview, focus group) is recommended |
| 3 Abstract | Summary of key elements of the study using the abstract format of the intended publication; typically includes objective, methods, results, and conclusions |
| **Introduction** |  |
| 4,5 Problem formulation | Description and significance of the problem/phenomenon studied; review of relevant theory and empirical work; problem statement |
| 4,5 Purpose or research question | Purpose of the study and specific objectives or questions |
| **Methods** |  |
| 5,6 Qualitative approach and research paradigm | Qualitative approach (e.g., ethnography, grounded theory, case study, phenomenology, narrative research) and guiding theory if appropriate; identifying the research paradigm (e.g., positivist, constructivist/interpretivist) is also recommended |
| 5,6 Researcher characteristics and reflexivity | Researchers’ characteristics that may influence the research, including personal attributes, qualifications/experience, relationship with participants, assumptions, or presuppositions; potential or actual interaction between researchers’ characteristics and the research questions, approach, methods, results, or transferability |
| 5,6 Context | Setting/site and salient contextual factors; rationale^a^ |
| 5,6 Sampling strategy | How and why research participants, documents, or events were selected; criteria for deciding when no further sampling was necessary (e.g., sampling saturation); rationale^a^ |
| 5,6 Ethical issues pertaining to human subjects | Documentation of approval by an appropriate ethics review board and participant consent, or explanation for lack thereof; other confidentiality and data security issues |
| 5,6 Data collection methods | Types of data collected; details of data collection procedures including (as appropriate) start and stop dates of data collection and analysis, iterative process, triangulation of sources/methods, and modification of procedures in response to evolving study findings; rationale^a^ |
| 5,6 Data collection instruments and technologies | Description of instruments (e.g., interview guides, questionnaires) and devices (e.g., audio recorders) used for data collection; if/how the instrument(s) changed over the course of the study |
| 5,6,7,8 Units of study | Number and relevant characteristics of participants, documents, or events included in the study; level of participation (could be reported in results) |
| 5,6,7 Data processing | Methods for processing data prior to and during analysis, including transcription, data entry, data management and security, verification of data integrity, data coding, and anonymization/deidentification of excerpts |
| 5,6,7,8 Data analysis | Process by which inferences, themes, etc., were identified and developed, including researchers involved in data analysis; usually references a specific paradigm or approach; rationale^a^ |
| 5,6,7,8 Techniques to enhance trustworthiness | Techniques to enhance trustworthiness and credibility of data analysis (e.g., member checking, audit trail, triangulation); rationale^a^ |
| **Results/Findings** |  |
| 9,10,11,12,13 Synthesis and interpretation | Main findings (e.g., interpretations, inferences, and themes); might include development of a theory or model, or integration with prior research or theory |
| 9,10,11,12,13 Links to empirical data | Evidence (e.g., quotes, field notes, text excerpts, photographs) to substantiate analytic findings |
| **Discussion** |  |
| 13,14,15,16,17 Integration with prior work, implications, transferability, and contribution(s) to the field | Short summary of main findings; explanation of how findings and conclusions connect to, support, elaborate on, or challenge conclusions of earlier scholarship; discussion of scope of application/generalizability; identification of unique contribution(s) to scholarship in a discipline or field |
| 16,17 Limitations | Trustworthiness and limitations of findings |
| **Other** |  |
| 18 Conflicts of interest | Potential sources of influence or perceived influence on study conduct and conclusions; how these were managed |
| 18 Funding | Sources of funding and other support; role of funders in data collection, interpretation, and reporting |

^a^The rationale should briefly discuss the justification for choosing that theory, approach, method, or technique rather than other options available, the assumptions and limitations implicit in those choices, and how those choices influence study conclusions and transferability. As appropriate, the rationale for several items might be discussed together.
